# The Effect of Different Thermal Treatment on the Allotropic fcc↔hcp Transformation and Compression Behavior of Polycrystalline Cobalt

**DOI:** 10.3390/ma13245775

**Published:** 2020-12-17

**Authors:** Michal Knapek, Peter Minárik, Patrik Dobroň, Jana Šmilauerová, Mayerling Martinez Celis, Eric Hug, František Chmelík

**Affiliations:** 1 Department of Physics of Materials, Faculty of Mathematics and Physics, Charles University, Ke Karlovu 5, 12116 Prague, Czech Republic; Peter.Minarik@mff.cuni.cz (P.M.); dobronp@karlov.mff.cuni.cz (P.D.); jana.smilauerova@gmail.com (J.Š.); mayerling.martinez@ensicaen.fr (M.M.C.); chmelik@met.mff.cuni.cz (F.C.); 2 Nuclear Physics Institute of the Czech Academy of Sciences, Husinec—Řež 130, 25068 Řež, Czech Republic; 3 Laboratoire de Cristallographie et Sciences des Matériaux, Normandie Université, CNRS UMR 6508, 6 Boulevard Maréchal Juin, 14050 Caen, France; eric.hug@ensicaen.fr

**Keywords:** cobalt, phase transformation, microstructure, deformation

## Abstract

Pure polycrystalline cobalt is systematically thermally treated in order to assess the effect of the microstructure on the compression behavior. Isothermal annealing of the as-drawn material leads to recrystallization and grain growth dependent on the annealing temperature (600–1100 ${}^{\circ }$C). Consequently, the yield strength decreases and the fracture strain increases as a function of rising grain size; the content of the residual fcc phase is ~6–11%. Subsequent thermal cycling around the transition temperature is applied to further modify the microstructure, especially in terms of the fcc phase content. With the increasing number of cycles, the grain size further increases and the fraction of the fcc phase significantly drops. At the same time, the values of both the yield strength and fracture strain somewhat decrease. An atypical decrease in the fracture strain as a function of grain size is explained in terms of decreasing fcc phase content; the stress-induced fcc→hcp transformation can accommodate a significant amount of plastic strain. Besides controlling basic material parameters (e.g., grain size and texture), adjusting the content of the fcc phase can thus provide an effective means of mechanical performance optimization with respect to particular applications.

## 1. Introduction

Metals with a hexagonal close-packed (hcp) crystal lattice belong to the prominent engineering materials used in the transport, aerospace, environmental, and information technology industries. For example, in the production of vehicles and airplanes, magnesium- and titanium-based materials deliver an excellent strength-to-weight ratio [1]. Furthermore, in the memory and data storage industries, miniaturized devices are being developed, which utilize cobalt and other hcp metals [2,3]. The design and reliability of such devices are essentially related to the understanding of their mechanical behavior, especially of the dynamics of the deformation mechanisms involved with respect to the material microstructure.

Pure cobalt features an almost ideal ratio of the lattice parameters (c/a), very low stacking fault energy, and a high melting point compared to other metals with an hcp structure [4]. hcp metals typically exhibit complex deformation dynamics due to only a limited number of slip systems, unlike face-centered cubic (fcc) metals [5]. Systematic studies of mechanical properties in relation to the microstructure of polycrystalline cobalt are, however, very scarce. Several existing studies mostly date back to the 1950s–1970s when the microstructural observations were still rather limited [6,7]. Other studies focused on cobalt thin films [8], micro-/nano-crystalline samples [9,10,11], or single crystals [6,12,13] or investigated the activity of various deformation modes including mechanical twinning mostly in tension [14,15,16,17,18].

Investigations of the strength-microstructure relation in polycrystalline cobalt are of particular interest because cobalt exhibits an allotropic hcp→fcc transition. The transition belongs to the class of martensitic phase transformations [19,20] and is rather sluggish as it is associated with a very low free-energy change of ~500 J/mol [4,21]. Martensitic transformation is a concept used currently in a relatively broad context, which describes diffusionless crystallographic transformations that are (almost) reversible. They are brought about by a movement of the interface between two phases as the atoms in the parent lattice realign into the more energetically favorable structure. During this first-order transformation, cooperative atomic shift results in crystal shape and symmetry changes [22]. Besides cobalt, martensitic transformations have been long known to occur in many metals and metallic alloys, such as Ti, steels, Ni-Ti alloys, and entropy alloys, frequently investigated using electron microscopy techniques [23,24,25].

In cobalt, the martensitic hcp→fcc transition takes place at a relatively low temperature of ~420 ${}^{\circ }$C. Upon cooling through this temperature, a significant fraction of the high-temperature fcc phase is typically retained in the microstructure (i.e., the reverse fcc→hcp transformation is incomplete). The aforementioned temperature is an average value of the martensite start and austenite start temperatures, which can exhibit a thermal hysteresis of up to 40 ${}^{\circ }$C in the case of polycrystalline cobalt [21]. It was shown that the fraction of the retained fcc phase in cobalt could be reduced by repeated thermal cycling around the transition temperature, thus providing an additional routine of microstructure tailoring [21,26,27,28]. Moreover, the residual fcc phase transforms to hcp modification upon loading at room temperature, thus making the deformation dynamics of cobalt even more complex [7,29]. Hence, the residual fcc phase stands as another microstructural parameter affecting the mechanical performance of the material, in addition to typical parameters such as grain size, dislocation density, crystallographic texture, etc.

Along these lines, the objective of this study is two-fold. Firstly, we systematically examine microstructure modifications and evolution in relation to the applied thermal treatments: isothermal annealing at different temperatures and thermal cycling in the vicinity of the hcp↔fcc transition. In particular, the recovery/recrystallization dynamics, grain growth, and fcc phase content evolution are examined in detail with the help of scanning electron microscopy and thermal analyses: thermodilatometry and differential scanning calorimetry. Secondly, we aim at assessing the microstructure-deformation behavior dependence, primarily focusing on the effect of the fcc phase content, which has been seldom investigated up until now. It is indeed shown that the fraction of the high-temperature fcc phase plays a significant role in the room-temperature deformation behavior of polycrystalline cobalt.

## 2. Materials and Methods

The polycrystalline cobalt was purchased from Goodfellow Cambridge Ltd. (Huntingdon, England) in the form of as-drawn rods (length of 200 mm, diameter of 6.35 mm). The purity of the material was 99.9%, having the following quoted content of impurities (in ppm): Fe—180, Ni—800, C—30, S—150. The rods were cut to obtain cylindrical samples with a height of 9 mm. The first set of samples was annealed using the vertical furnace Nabertherm RHTV 120-600 (Lilienthal, Germany) under vacuum for 1 h at the temperatures of 600–1100 ${}^{\circ }$C (100 ${}^{\circ }$C step) and water-quenched. The second and third set of samples were subjected to the same annealing procedure and subsequently thermally cycled (second set—10 cycles, third set—20 cycles). One thermal cycle consisted of linear heating from 300 to 550 ${}^{\circ }$C and subsequent cooling down to 300 ${}^{\circ }$C, both at a rate of 5 ${}^{\circ }$C/min. Thermal cycling was performed using the Linseis L75 PT vertical thermodilatometer (Selb, Germany) under an Ar atmosphere (for the thermodilatometric (TD) data used in the paper, correction curves were subtracted). Differential scanning calorimetry (DSC) was carried out using the Netzsch DSC 404 C Pegasus apparatus (Selb, Germany) under vacuum with the same thermal cycling program. For DSC measurements, small discs were cut from the samples and mechanically polished using 4000 grit paper to obtain a mass of ~30 mg.

Microstructural changes in the investigated samples were studied by electron backscatter diffraction (EBSD). The samples for EBSD were prepared by mechanical grinding by emery papers and polishing using diamond suspensions with particle size decreasing down to 1 μm. Subsequently, the final ion-etching was performed by Leica EM RES102 (Leica Mikrosysteme, Wetzlar, Germany). The measurements were performed in the scanning electron microscope (SEM) ZEISS Auriga Compact FIB-SEM (Jena, Germany) equipped with the EDAXEBSD camera (Berwyn, PA, USA). The EBSD study was focused (i) on the volume fraction of the residual fcc phase and (ii) on the grain size and texture of the hcp phase. The observations regarding (i) were performed on the maps showing an area of 400 × 400 μm${}^{2}$ collected with a step size of 0.4 μm, while the analyses concerning (ii) were performed on EBSD maps with an area of 1000 × 2000 μm${}^{2}$ and with a step size of 1.5 μm. Such a variation in the scan size was crucial because of the significant difference in the sizes of fcc and hcp phase grains. Subsequently, the measured data were partially cleaned using the EDAX OIM TSL 7 software by one step of confidence index (CI) standardization, one step of phase neighbor correlation, and one iteration of grain dilatation. Only points having a CI higher than 0.1 were used for further analysis. The average grain size was calculated for areas separated by grain boundaries with a misorientation higher than 15${}^{\circ }$ [30], as a weighted average with the area fraction as the weight. Inverse pole figures (IPF) were generated from the EBSD data by harmonic series expansion up to the rank of 16.

The compression tests on the samples (height of 9 mm, diameter of 6.35 mm) of each set were performed using the Instron 5582 (Norwood, MA, USA) universal testing machine at room-temperature and the initial strain rate of 10${}^{-3}\;\cdot \;$s${}^{-1}$. The machine stiffness was subtracted from the deformation data before further processing. Apiezon M grease was used as a lubricant to suppress barreling of the samples. The systematic experimental error was determined to be 3%. Several tests were repeated in order to verify the consistency of the results.

The following naming convention is used throughout the paper: AD—initial (as-drawn) samples, (600–1100)—samples annealed for 1 h at 600–1100 ${}^{\circ }$C, and (600-*xx*c–1100-*xx*c)—samples annealed for 1 h at 600–1100 ${}^{\circ }$C and subjected to the thermal cycling of *xx* cycles.

## 3. Results

**Figure 1:**
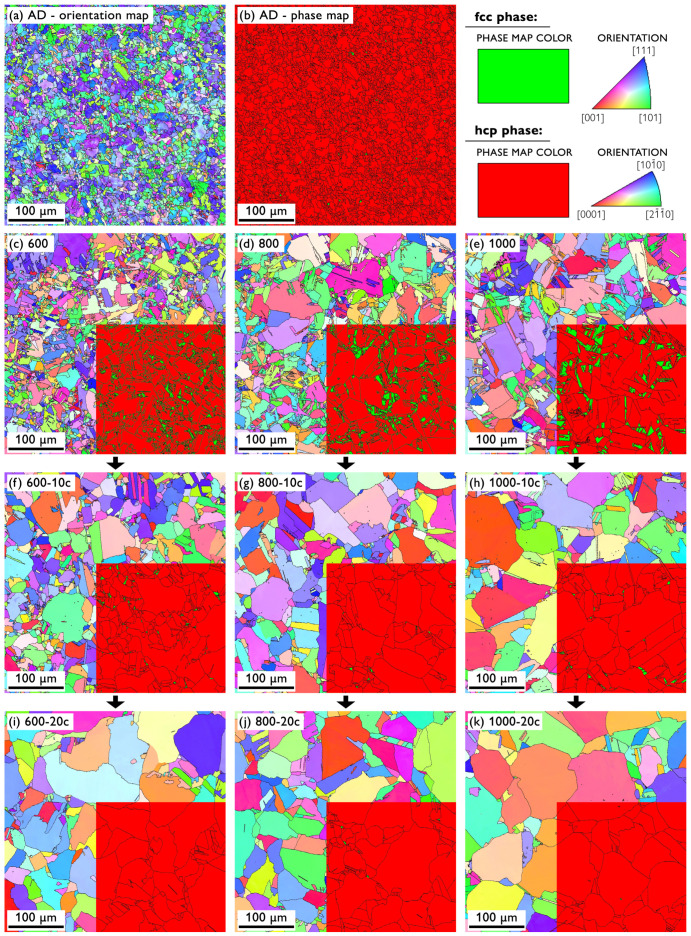
SEM EBSD micrographs of the selected samples before the thermal treatment: (**a**) AD—orientation map, (**b**) AD—phase map; and after the thermal treatment: (**c**–**e**) samples 600, 800, and 1000, respectively; (**f**–**h**) samples 600-10c, 800-10c, and 1000-10c, respectively; (**i**–**k**) samples 600-20c, 800-10c, and 1000-20c, respectively. The parent images of the samples (c–k) represent orientation maps and the insets represent phase maps of the respective part of each image. The processing direction (i.e., the rod lengthwise direction during drawing) is perpendicular to the paper plane.

The microstructural features of the initial as-drawn samples and the sample sets subjected to different thermal treatments were examined in detail. The EBSD micrographs of the selected samples, showing the orientation maps, as well as the phase maps, are presented in Figure 1. The AD sample featured a deformed (see the color variations within a single grain) and relatively fine microstructure with a mean grain size of ~9 μm and less than 2% of the residual fcc phase (see also Table 1). Isothermal annealing of the initial AD material for 1 h led to an increase in the grain size to 22–46 μm with apparent dependence on the annealing temperature, as observed in Figure 1c–e, demonstrating the occurrence of recovery, recrystallization, and grain growth (for the values, see also Table 1). At the same time, the amount of the residual fcc phase increased to ~6–11%, with no clear dependence on the annealing temperature. The samples annealed at different temperatures were subsequently subjected to thermal cycling in the temperature range of 300–550 ${}^{\circ }$C, i.e., the range within which the hcp↔fcc allotropic phase transformation takes place. Ten such thermal cycles resulted in further grain growth (to ~30–60 μm), still reflecting the differences in the microstructure after isothermal annealing (Figure 1f–h). Concurrently, the fraction of the fcc phase decreased to ~1% for the sample 600 and below 0.5% for the samples 700–1100. Finally, after 20 thermal cycles, the grain growth was further promoted, achieving a grain size of ~60 μm, and the fraction of the fcc phase was reduced below 0.5% for all the samples (Figure 1i–k and Table 1).

**Figure 2:**
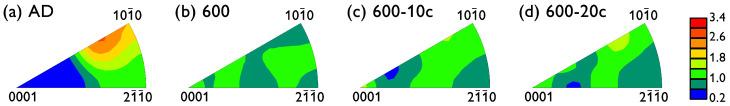
EBSD inverse pole figures of the selected samples: (**a**) AD sample, (**b**) sample 600, (**c**) sample 600-10c, and (**d**) sample 600-20c. The intensity scale bar (multiples of a random distribution) is valid for all the images.

Figure 2 shows the crystallographic texture by means of inverse pole figures of the selected samples: AD, 600, 600-10c, and 600-20c. A certain texture can be observed for the AD sample, comprising the components typical of drawn or extruded rods of hcp metals [31]. This rather weak texture, however, vanished already during isothermal annealing at the lowest temperature of 600 ${}^{\circ }$C (sample 600, Figure 2b). Additional thermal treatment of the sample 600 by means of thermal cycling did not bring about any perceptible changes in the texture, which remained rather random and very feeble. The other samples not shown in Figure 2 exhibited a similar or even weaker crystallographic texture, in line with the observed random EBSD orientation maps in Figure 1. Note that the texture was calculated from much larger areas than shown in Figure 1, comprising a sufficient number of grains (see the Materials and Methods).

**Figure 3:**
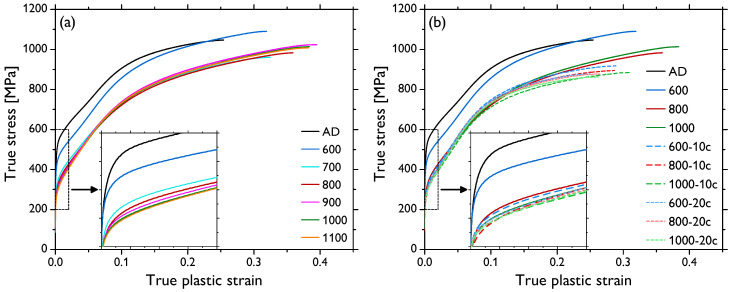
Deformation curves of the selected samples: (**a**) AD sample and samples 600–1000, (**b**) AD sample and samples 600, 800, and 1000 (for the sake of comparison), samples 600-10c, 800-10c, 1000-10c, and samples 600-20c, 800-20c, 1000-20c.

The compression tests were performed on each sample type, and the selected deformation curves are presented in Figure 3; the insets show the details of the elasto-plastic transition. In Figure 3a, the compression curves of the annealed samples, 600–1100, are depicted, demonstrating (i) an increasing fracture strain ($\varepsilon_{\max}$) of the samples (from ~0.25 for AD to ~0.38 for 900, 1000, and 1100), (ii) a gradually declining yield strength $\sigma_{0.2}$ of the samples (from ~486 MPa for AD down to ~269 MPa for 1100), and (iii) a non-monotonous evolution of the compressive strength ($\sigma_{\max}$) of the samples, ranging from ~960 to ~1090 MPa, as a function of increasing annealing temperature (see also Table 1, listing all the data). Figure 3b depicts several reference deformation curves from Figure 3a (samples AD, 600, 800, and 1000) and the deformation curves of the respective samples subjected to the thermal cycling of 10 cycles (samples 600-10c, 800-10c, and 1000-10c) and 20 cycles (samples 600-20c, 800-20c, and 1000-20c). It was observed that the thermal cycling of 10 cycles resulted in a decrease in all the values of evaluated mechanical parameters—$\varepsilon_{\max}$, $\sigma_{\max}$, and $\sigma_{0.2}$—giving the values in the relatively narrow ranges of ~0.27–0.32, ~885–926 MPa, and ~257–287 MPa, respectively. No obvious dependence of these values on the temperature of the preceding isothermal annealing was observed, even though some trend was still noticed in the grain size values (Table 1). After the thermal cycling of 20 cycles, the values of all the mechanical parameters—$\varepsilon_{\max}$, $\sigma_{\max}$, and $\sigma_{0.2}$—exhibited a further slight decrease to ~0.25–0.28, ~863–885 MPa, and ~254–276 MPa, respectively, again with no clear trends in relation to the thermal history (see Table 1).

**Figure 4:**
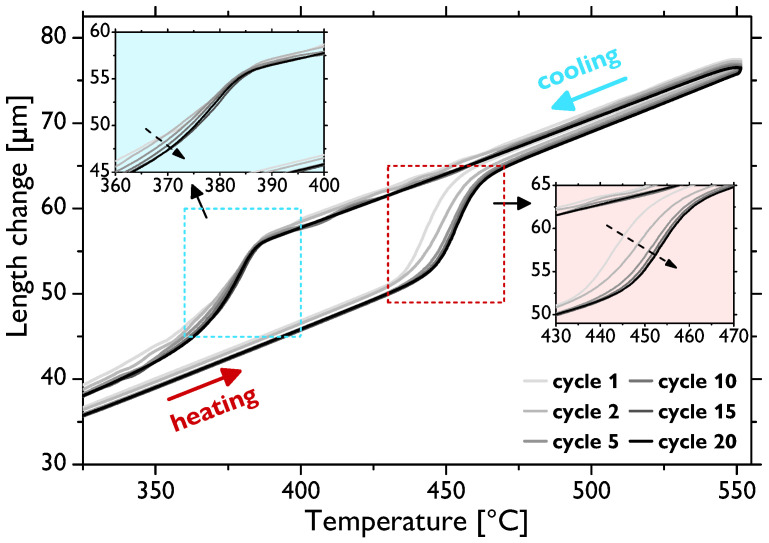
Selected thermodilatometric (TD) curves of the sample 1000 subjected to thermal cycling of 20 cycles.

**Figure 5:**
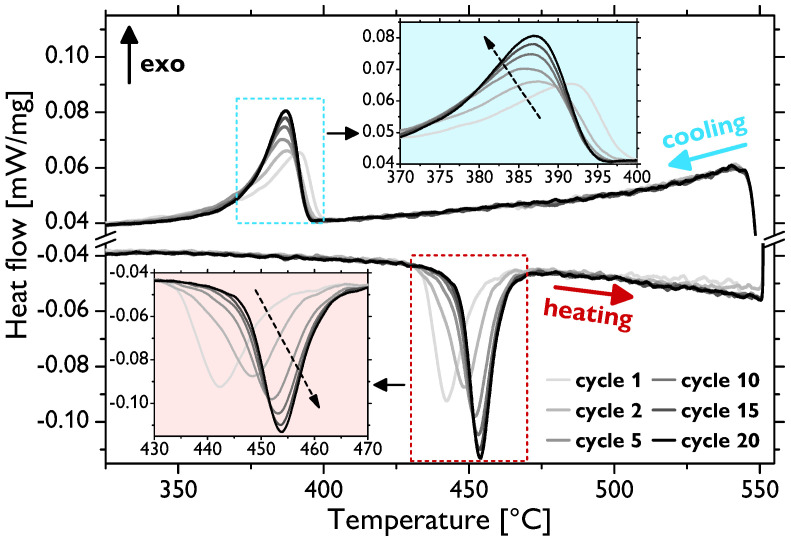
Selected DSC curves of the sample 1000 subjected to thermal cycling of 20 cycles.

**Figure 6:**
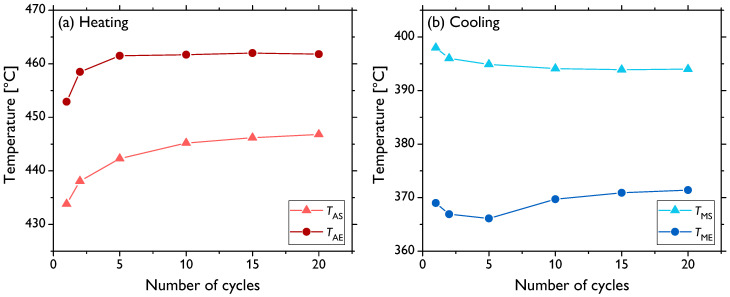
Evolution of the $T_{\mathrm{AS}}$, $T_{\mathrm{AE}}$, $T_{\mathrm{MS}}$, and $T_{\mathrm{ME}}$ temperatures determined from the DSC peaks as a function of the increasing number of thermal cycles (**a**) heating, (**b**) cooling.

As presented above, besides the evolution of the grain size and mechanical properties, the fraction of the residual fcc phase was another significantly affected quantity depending on different types of thermal treatment. The reversible hcp↔fcc transition is a martensitic-type transformation [4,32] and, thus, can be effectively investigated by conventional thermal analyses. For such observations, sample 1000 (i.e., the sample annealed for 1 h at 1000 ${}^{\circ }$C) was used, as its microstructure was already fairly recrystallized, hence enabling the sole investigation of the fcc phase evolution without a significant influence of the evolution of other microstructural features. Figure 4 shows the thermodilatometric (TD) data recorded during thermal cycling. For the sake of clarity, only selected cycles—Cycles 1, 2, 5, 10, 15, and 20—are presented. Most portions of the curves exhibit quasi-linear dependence on the temperature, which is the typical behavior of pure metals. However, there are gradual jumps in this linear behavior from around 450 to 470 ${}^{\circ }$C during the heating and from about 380 to 350 ${}^{\circ }$C during the cooling stages of cycling related to the volume changes associated with the transition (the maximum theoretical volume difference was ~0.3–0.4% [7,33]). These temperatures thus correspond to the austenite start ($T_{\mathrm{AS}}$), austenite end ($T_{\mathrm{AE}}$), martensite start ($T_{\mathrm{MS}}$), and martensite end ($T_{\mathrm{ME}}$) temperatures, respectively. The observed temperature intervals and thermal hysteresis agreed well with the previously reported kinetic analyses of the hcp↔fcc transition in pure cobalt [21,34], as will be discussed in detail later. It is also evident that the shape of the curves in the vicinity of the transition evolves with an increasing number of cycles (for details, see Figure 4 insets). The $T_{\mathrm{AS}}$ temperature successively shifts to slightly higher values, whereas, on the other hand, $T_{\mathrm{MS}}$ seems to be not affected. The $T_{\mathrm{AE}}$ and $T_{\mathrm{ME}}$ temperatures cannot be directly distinguished from the measured TD data, which are alone difficult to use for quantitative analysis and, especially, for the precise determination of the transition temperatures. To that end, differential scanning calorimetry (DSC) analysis was employed maintaining the same conditions (thermal cycling program) and sample type (1000). Figure 5 presents the data of the selected cycles as in the case of TD. There are well-defined peaks corresponding to the hcp↔fcc transition during the heating and cooling stages of each cycle. The peak analysis allowed for quite accurate calculations of the transition temperatures by means of finding the intersections of the baselines and the tangent lines at the peak left and right inflection points. The evolution of the thus-determined temperatures is shown in Figure 6. These data show that, indeed, the entire endothermic austenitic peak (i.e., both $T_{\mathrm{AS}}$ and $T_{\mathrm{AE}}$) shifts to a somewhat higher temperature as the number of cycles increases (to be precise, the $T_{\mathrm{AE}}$ value is rather saturated already after five cycles). Moreover, the temperature difference between $T_{\mathrm{AS}}$ and $T_{\mathrm{AE}}$ slightly decreases (Figure 6a) as the peak sharpens and its height increases (Figure 5). The evolution of the transition temperatures of the exothermic martensitic peak, $T_{\mathrm{MS}}$ and $T_{\mathrm{ME}}$, is less straightforward. The $T_{\mathrm{MS}}$ temperature gradually decreases throughout the entire cycling; however, $T_{\mathrm{ME}}$ decreases only up to five cycles and subsequently starts to rise (Figure 6b). Therefore, this cooling peak also narrows during cycling (the latter stages) and its height increases, in agreement with the observations for the heating peak. The cooling peak is, however, less intense, and the martensitic fcc↔hcp transformation manifestly proceeds slower than the austenitic (hcp↔fcc) one, this being well visible in Figure 5 and also demonstrated by the higher temperature difference in Figure 6b compared to Figure 6a. Nevertheless, it can be clearly inferred that the thermal hysteresis ($\Delta T=T_{\mathrm{AS}}-T_{\mathrm{MS}}$) increases with a rising number of thermal cycles. Fairly similar shapes and the evolution of the DSC peak in cobalt associated with the hcp↔fcc transition were observed elsewhere [21,26,34].

## 4. Discussion

The mechanical properties of crystalline materials are, apart from the chemical composition, critically affected by their microstructural features. The flow stress and deformability of a material are governed primarily by the grain size (through the Hall–Petch relation), the Taylor strengthening related to the dislocation density (i.e., associated with the residual strain), the solid solution strengthening, and the precipitation strengthening, out of which the latter two can be neglected in this study as the impurity content was very low in the cobalt samples. In addition to these four basic strengthening mechanisms, the proportion of different allotropic phases contained in pure metallic polycrystals, which exhibit allotropic transformation involving a relatively low thermodynamic driving force for the transition, can be of high significance.

**Figure 7:**
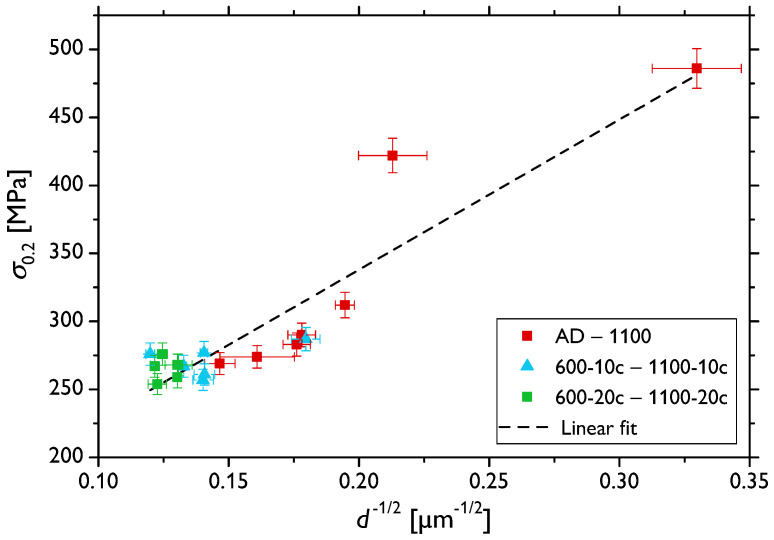
The Hall–Petch plot for all the investigated samples sets—initial sample, annealed samples, and thermally-cycled samples.

Softening due to the increase in the grain size was observed in the investigated samples, as seen from the comparison of Figure 1 and Figure 3 and, especially, in Table 1. This dependence is also confirmed by means of the conventional Hall–Petch plot shown in Figure 7 with points calculated for all the sample types involved in this study. This graph, however, does not show a perfectly linear behavior of the yield strength, $\sigma_{0.2}$, as a function of the reciprocal of the square root of grain size, $d^{-\frac{1}{2}}$, as predicted by the theory for such intermediate grain sizes [17,35,36]. The deviations might be brought about by several factors: (i) there was a very limited number of samples with a small grain size (since the applied heat treatment and cycling led to a rather expeditious grain growth) to ensure better statistics; (ii) besides the grain growth, the initially deformed microstructure, as demonstrated by color variations within individual grains in Figure 1a, recovered and recrystallized (Figure 1c–k); and (iii) the fraction of the residual fcc phase significantly decreased due to the heat treatment. Nevertheless, there was a clear dependence of $\sigma_{0.2}$ for the samples AD and 600–1100 marked in red, which exhibited distinct grain size variations (see also the inset in Figure 3a), in agreement with previously reported observations [7]. On the other hand, the thermally cycled sample sets (600-10c to 1100-10c and 600-20c to 1100-20c marked in blue and green, respectively) featuring much narrower ranges of larger recrystallized grains rather formed a “cluster”, with no clear grain size effect on the $\sigma_{0.2}$ value.

The dependence of the compressive strength ($\sigma_{\max}$) and fracture strain ($\varepsilon_{\max}$) values was more straightforward in some respects. The sample sets AD and 600–1100 exhibited a clear positive dependence of $\varepsilon_{\max}$ on the grain size (which itself depended positively on the annealing temperature), as it gradually increased from ~0.25 (AD) to ~0.38 (1100), this being a typical behavior of cobalt and metallic polycrystals in general [7]. Yet, very similar $\sigma_{\max}$ values of ~1000 MPa were found for all the samples within this set, independent of the grain size. Another direct observation is the decrease in both the $\varepsilon_{\max}$ and $\sigma_{\max}$ values due to the thermal cycling, whereas the values were rather constant across the samples of the same set—$\varepsilon_{\max}$ = ~0.27, $\sigma_{\max}$ = ~900 MPa after 10 cycles and $\varepsilon_{\max}$ = ~0.30, $\sigma_{\max}$ = ~880 MPa after 20 thermal cycles (cf. Figure 3 and Table 1). Decreasing $\sigma_{\max}$ as a result of thermal cycling can be readily related to the microstructure evolution, i.e., the recovery, recrystallization, and grain growth (the grain growth in cobalt is typically promoted when temperatures above 500 ${}^{\circ }$C during cycling are involved [7]). One would, however, expect that the recrystallized microstructure and larger grain size (after 20 thermal cycles as opposed to 10) also would give rise to higher deformability ($\varepsilon_{\max}$), which was not the case here. In order to elucidate this observation, we must finally take into account the content of the residual fcc phase, which was shown to substantially affect the deformability of cobalt and the overall shape of its deformation curves [7]. During loading of polycrystalline cobalt samples containing the fcc phase, deformation-induced martensitic fcc↔hcp transformation takes place, contributing to the sample ductility. The driving force for such stress-induced transition is actually orders of magnitude higher than that during the thermal cycling [7]. Hence, these results strongly suggest that not only the grain size, but also the fcc phase content might be the determining factor of the sample deformability. It should be, however, noted that even though there were observable differences in the fcc fraction between the samples subjected to 10 and 20 thermal cycles (cf. Figure 1), its content was already rather low (<1%) in both sets. Still, the most significant decrease in the $\varepsilon_{\max}$ value occurred after the thermal cycling (in comparison with the annealed samples 600–1100), in accordance with a rather significant reduction in the residual fcc phase (cf. Table 1). The theoretical volume change of ~0.3–0.4% can accommodate only a very limited strain. However, it was asserted that due to the abundance of slip systems in the fcc phase, the slip and transformation are closely related and may bring about grain shape change, thus providing large amounts of shear [7]. It was estimated that in this way, a strain of 0.25% can be theoretically attained by a full transformation of 1% of the retained fcc phase. The deformation-induced transformation in cobalt is, however, typically completed by a strain of roughly 10% (depending on the materials and testing conditions [7,29]), and in the latter stages of deformation, mechanical twinning takes place as the secondary deformation mechanism [7,18]. Indeed, it was documented that only basal slip is typically active in cobalt with no prominent secondary slip systems available, while a multiplicity of twinning systems can be activated [7]. It is also believed that in hcp metals, twinning is more readily activated in materials with larger grains [31,37]. The formation of twins gives rise to the abundance of high-angle grain boundaries, which, in turn, strengthen the material and make it less ductile (since these new boundaries act as barriers to dislocation slip, notwithstanding that the slip can be to some extent also promoted due to crystal reorientation). In this manner, the ductility of the material featuring larger grains can be even lower than in the case of materials with smaller grains [38,39]. We speculate that such an effect can be present also in the investigated cobalt samples and, together with the influence of the residual fcc phase, can account for the observed reduced fracture strain ($\varepsilon_{\max}$) observed despite the microstructure recovery, recrystallization, and grain growth. To shed more light on this matter, quantitative (semi) in situ microstructural observations are needed, those being the subject of the ensuing separate study.

The dynamics of the hcp↔fcc, as evaluated by means of thermal analyses (Figure 4, Figure 5 and Figure 6), also confirms that the microstructure is not stable after 10 cycles and the changes in the TD and DSC curves, even though they somewhat slow down, continue to take place up to 20 cycles. Indeed, it was shown that a completely stable microstructure of polycrystalline cobalt can be obtained only after more than ~50 cycles around the transition temperature (although the exact evolution arguably depends on the conditions, such as initial microstructure, temperature range, etc.) [21,26,28]. Therefore, notwithstanding that the content of the fcc phase is very low after both 10 and 20 cycles, further evolution of the microstructure occurs, as was also discernible in the (rather local) EBSD images in Figure 1.

In order to comprehend the evolution of the microstructure during thermal cycling, especially in relation to the phase transformation, the calorimetric curves must be examined in detail. Sharpening and intensification of both the endothermic and exothermic peaks (i.e., during heating and cooling stages, respectively) with an increasing number of thermal cycles signifies acceleration of the hcp↔fcc transition. It was also documented in [21] that the $T_{\mathrm{AS}}$, $T_{\mathrm{AE}}$, $T_{\mathrm{MS}}$, and $T_{\mathrm{ME}}$ temperatures tend to shift, and at the same time, the shape of the peaks changes during cycling. The thermal hysteresis, i.e., the difference between the transformation temperatures upon heating and cooling, increases with thermal cycling (apart from some slight deviations in $T_{\mathrm{ME}}$). This effect arises due to the strain energy induced by volume changes and the reduction in the number of nucleation sites during cycling, as will be discussed below [6,40].

It was shown as early as in the 1970s that the hcp↔fcc transition proceeds to completion only rarely, e.g., when a single interface transformation takes place in single crystals [6]. Several theories have been proposed to account for the incomplete fcc→hcp transition upon cooling through the $T_{\mathrm{MS}}$ and $T_{\mathrm{ME}}$ temperatures. They involved the passage of Shockley partial dislocations (SPs) over every second closest-packed plane {111} in the fcc lattice to form the hcp structure, and these theories differ only in the mechanisms of dislocation generation and movement [41,42,43]. Much later, these theories were refined, and the following explanation summarized, e.g., by Bauer et al. [21] is currently rather generally accepted. The fcc↔hcp transition (i.e., the transformation from the ABCABC … to ABABAB… stacking sequence and back) is realized by an ordered glide of SPs with $\frac{1}{6}\langle 11\bar{2}\rangle$ Burgers vectors on every second {111} plane. The fcc→hcp transition takes place by dissociation of the perfect dislocations into SPs and the hcp→fcc one by association of the same SPs (i.e., by a reverse dislocation reaction). During the first thermal cycles, entire grains cannot fully transform back to the hcp structure due to mutual blocking of the growing martensite along one of the four equivalent {111} fcc planes. Continued thermal cycling, however, leads to the preferred growth along favorably oriented planes by progressively seizing the nearby dislocations. As the hcp structure has only a single closest-packed set of {0001} planes and the fcc structure features four equivalent sets of {111} planes, the dislocation structure gradually rearranges so that only one active {111} set parallel to the {0001} plane remains finally active (see also the comprehensive schematic views in [21]). In other words, the first cycles involve the rearrangement of crystal defects, while several tens of cycles are needed to facilitate a fully reversible hcp↔fcc transition. Along these lines, it is not surprising that there were more significant changes in the TD (Figure 4) and DSC data (Figure 6) in the course of the initial thermal cycles, whereas the process gradually stabilized with the increasing number of cycles. Yet, it might then seem counterintuitive at first glance why the thermal hysteresis becomes larger during thermal cycling. In fact, this delay in the transformation also interrelates with the microstructure evolution; however, different processes are responsible for that effect. Specifically, transformation-induced defects (mostly sessile dislocations), which are effectively generated during thermal cycling, interact with the above-described interface dislocations. The expanded hysteresis corresponds to the amount of energy needed to overcome these pinned transformation-induced defects during subsequent cycles [26].

The understanding of the physical mechanisms underlying the dynamics of the hcp↔fcc transition can be thus effectively utilized for fine-tuning the material microstructure, e.g., via thermo-mechanical treatment. In turn, the mechanical performance of polycrystalline cobalt, which was shown to be significantly affected by the content of the retained high-temperature fcc phase, can be systematically optimized to meet the application-specific requirements.

## 5. Conclusions

Pure polycrystalline cobalt was thermally treated in order to systematically modify its microstructure and, in turn, to examine its effect on the mechanical properties in compression. The main conclusions drawn from this study can be summarized as follows:

- The initial as-drawn samples feature a deformed and relatively fine microstructure with a grain size of ~9 μm. Isothermal annealing for one hour at different temperatures in the range of 600–1100 ${}^{\circ }$C leads to grain growth, yielding grain sizes ranging from ~22 μm to ~47 μm with explicit dependence on the annealing temperature. Consequently, the yield strength ($\sigma_{0.2}$) drops from ~486 MPa down to ~269 MPa and the fracture strain ($\varepsilon_{\max}$) increases from ~0.25 to ~0.38 as a function of rising grain size. There are, however, no straightforward trends observed in the compressive strength ($\sigma_{\max}$) with increasing grain size, having the values of ~961–1091 MPa. The content of the residual high-temperature fcc phase is ~6–11% across this sample set.
- Subsequent thermal cycling in the temperature range of 300–550 ${}^{\circ }$C (10 or 20 cycles) is applied to assess the evolution of the microstructure and mechanical performance. After 10 cycles, the grain size further increases to ~31–70 μm, still somewhat reflecting the microstructure differences after the preceding annealing at various temperatures. On the other hand, no clear dependencies on the grain size of $\varepsilon_{\max}$, $\sigma_{\max}$, and $\sigma_{0.2}$ are observed, and their values are ~0.27–0.32, ~885–926 MPa, and ~257–287 MPa, respectively. The content of the fcc phase drops to below 1%. After 20 cycles, the grain size of ~60 μm is evidenced for all the samples. The $\varepsilon_{\max}$, $\sigma_{\max}$, and $\sigma_{0.2}$ values decrease slightly further to ~0.25–0.28, ~863–885 MPa, and ~254–276 MPa, respectively. The fraction of the fcc phase drops below 0.5%.
- An atypical decrease in $\varepsilon_{\max}$ and ambiguous trends in the other mechanical quantities as a function of grain size are explained in terms of decreasing fcc phase content upon thermal cycling. Upon loading, the fcc phase exhibits a stress-induced fcc→hcp transformation, thus accommodating a significant amount of plastic strain (by the associated volume change and by providing additional slip systems).
- The processes involved in the hcp↔fcc transformation during thermal cycling are studied by differential scanning colorimetry, and it is evidenced that a complete transformation does not occur even after 20 cycles. Yet, the effectiveness of the transformation improves with an increasing number of cycles owing to the evolving dislocation structure, which plays a crucial role in the transformation dynamics.
- Apart from modifying the standard microstructural features, such as grain size and dislocation density, tailoring the content of the residual fcc phase seems to be crucial for attaining the desired mechanical performance of polycrystalline cobalt. Such an approach may, therefore, open new possibilities for effective mechanical performance optimization with respect to particular applications.

## Figures and Tables

**Table 1:** Microstructural and mechanical data of all the tested sample sets.

Sample	Grain Size (μm)	fcc Fraction (%)	$\varepsilon_{\max}$	$\sigma_{\max}$ (MPa)	$\sigma_{0.2}$ (MPa)
**AD**	9 ± 1	~2	0.25 ± 0.01	1047 ± 31	486 ± 15
**600**	22 ± 3	~6	0.32 ± 0.01	1091 ± 33	422 ± 13
**700**	26 ± 1	~10	0.33 ± 0.01	961 ± 29	312 ± 9
**800**	32 ± 2	~8	0.36 ± 0.01	984 ± 30	290 ± 9
**900**	32 ± 2	~11	0.39 ± 0.01	1024 ± 31	283 ± 8
**1000**	39 ± 8	~11	0.38 ± 0.01	1014 ± 30	274 ± 8
**1100**	47 ± 4	~6	0.38 ± 0.01	1008 ± 30	269 ± 8
**600-10c**	31 ± 2	~1	0.29 ± 0.01	918 ± 28	287 ± 9
**700-10c**	50 ± 1	<0.5	0.32 ± 0.01	926 ± 28	277 ± 8
**800-10c**	51 ± 3	<0.5	0.29 ± 0.01	895 ± 27	257 ± 8
**900-10c**	51 ± 3	<0.5	0.27 ± 0.01	887 ± 27	261 ± 8
**1000-10c**	57 ± 1	<0.5	0.31 ± 0.01	885 ± 27	267 ± 8
**1100-10c**	70 ± 2	<0.5	0.32 ± 0.01	894 ± 27	276 ± 8
**600-20c**	65 ± 2	<0.5	0.25 ± 0.01	879 ± 26	276 ± 8
**700-20c**	59 ± 5	<0.5	0.28 ± 0.01	885 ± 27	268 ± 8
**800-20c**	59 ± 1	<0.5	0.26 ± 0.01	878 ± 26	268 ± 8
**900-20c**	59 ± 1	<0.5	0.28 ± 0.01	879 ± 26	259 ± 8
**1000-20c**	68 ± 2	<0.5	0.26 ± 0.01	863 ± 26	267 ± 8
**1100-20c**	67 ± 4	<0.5	0.27 ± 0.01	864 ± 26	254 ± 8
